# Role of LmeA, a Mycobacterial Periplasmic Protein, in Maintaining the Mannosyltransferase MptA and Its Product Lipomannan under Stress

**DOI:** 10.1128/mSphere.01039-20

**Published:** 2020-11-04

**Authors:** Kathryn C. Rahlwes, Sarah H. Osman, Yasu S. Morita

**Affiliations:** a Department of Microbiology, University of Massachusetts, Amherst, Massachusetts, USA; University of Iowa

**Keywords:** *Mycobacterium*, biosynthesis, glycolipids, mannose, stress response

## Abstract

The mycobacterial cell envelope has a diderm structure, composed of an outer mycomembrane, an arabinogalactan-peptidoglycan cell wall, a periplasm, and an inner membrane. Lipomannan (LM) and lipoarabinomannan (LAM) are structural and immunomodulatory components of this cell envelope. LM/LAM biosynthesis involves a number of mannosyltransferases and acyltransferases, and MptA is an α1,6-mannosyltransferase involved in the final extension of the mannan chain. Recently, we reported the periplasmic protein LmeA being involved in the maturation of the mannan backbone in Mycobacterium smegmatis. Here, we examined the role of LmeA under stress conditions. We found that *lmeA* transcription was upregulated under two stress conditions: stationary growth phase and nutrient starvation. Under both conditions, LAM was decreased, but LM was relatively stable, suggesting that maintaining the cellular level of LM under stress is important. Surprisingly, the protein levels of MptA were decreased in an *lmeA* deletion (Δ*lmeA*) mutant under both stress conditions. The transcript levels of *mptA* in the Δ*lmeA* mutant were similar to or even higher than those in the wild type, indicating that the decrease of MptA protein was a posttranscriptional event. The Δ*lmeA* mutant was unable to maintain the cellular level of LM under stress, consistent with the decrease in MptA. Even during active growth, overexpression of LmeA led the cells to produce more LM and become more resistant to several antibiotics. Altogether, our study reveals the roles of LmeA in the homeostasis of the MptA mannosyltransferase, particularly under stress conditions, ensuring the stable expression of LM and the maintenance of cell envelope integrity.

**IMPORTANCE** Mycobacteria differentially regulate the cellular amounts of lipoglycans in response to environmental changes, but the molecular mechanisms of this regulation remain unknown. Here, we demonstrate that cellular lipoarabinomannan (LAM) levels rapidly decline under two stress conditions, stationary growth phase and nutrient starvation, while the levels of another related lipoglycan, lipomannan (LM), stay relatively constant. The persistence of LM under stress correlated with the maintenance of two key mannosyltransferases, MptA and MptC, in the LM biosynthetic pathway. We further showed that the stress exposures lead to the upregulation of *lmeA* gene expression and that the periplasmic protein LmeA plays a key role in maintaining the enzyme MptA and its product LM under stress conditions. These findings reveal new aspects of how lipoglycan biosynthesis is regulated under stress conditions in mycobacteria.

## Abstract (precis)

Mycobacteria differentially regulate the cellular amounts of lipoglycans in response to environmental changes, but the molecular mechanisms of this regulation remain unknown. Here, we demonstrate that cellular lipoarabinomannan (LAM) levels rapidly decline under two stress conditions, stationary growth phase and nutrient starvation, while the levels of another related lipoglycan, lipomannan (LM), stay relatively constant. The persistence of LM under stress correlated with the maintenance of two key mannosyltransferases, MptA and MptC, in the LM biosynthetic pathway. We further showed that the stress exposures lead to the upregulation of *lmeA* gene expression and that the periplasmic protein LmeA plays a key role in maintaining the enzyme MptA and its product LM under stress conditions. These findings reveal new aspects of how lipoglycan biosynthesis is regulated under stress conditions in mycobacteria.

## INTRODUCTION

Mycobacterium tuberculosis is the leading cause of death due to a bacterial pathogen, claiming 1.2 million deaths in 2018 alone (1). Furthermore, nearly half a million new cases of multidrug-resistant M. tuberculosis were reported (1), indicating an urgent need to discover novel drug targets. Mycobacteria have one of the most impermeable cell envelopes of all bacteria due to its waxy surface, creating an effective barrier against antibiotics and host immune attacks. In a currently proposed model, the outer membrane (OM) is made of an outer leaflet composed of various (glyco)lipids and an inner leaflet composed primarily of mycolic acids. These mycolic acids are linked to arabinan in the arabinogalactan (AG) layer. This layer in turn is covalently attached to the peptidoglycan (PG) cell wall (2–6).

Phosphatidylinositol (PI) mannosides (PIMs), lipomannan (LM), and lipoarabinomannan (LAM) are mannose-rich molecules in which the mannose chain is linked to a PI membrane anchor. PIMs are glycolipids that carry up to six mannose residues. They are suggested to be components of the plasma membrane (7, 8), playing both structural and functional roles. For example, a lack of hexamannosyl PIM species, such as AcPIM6 and Ac_2_PIM6 in Mycobacterium smegmatis, results in plasma membrane invaginations, a growth delay, an increase in antibiotic sensitivity, and a defective permeability barrier (8, 9). LM and LAM are lipoglycans much larger than PIMs, carrying more than 20 mannose residues. These lipoglycans are thought to be present in both the inner membrane and the OM of mycobacteria (2) and appear to play a structural role in cell envelope integrity (10). In M. tuberculosis, LAM is important for the effective establishment of infection in mice and interacts with several host receptors, such as Dectin-2, DC-SIGN, the mannose receptor, and lactosylceramide lipid rafts (11–14). Furthermore, changes in the mannan chain length of LM/LAM decrease the virulence of M. tuberculosis in mice (10).

The biosynthesis of PIMs/LM/LAM starts with PI. Two mannoses are added from GDP-mannose by sequential actions of two mannosyltransferases, PimA and PimB′, forming PIM2. One or two acyl chains are then added, forming AcPIM2 and Ac_2_PIM2 (15–17). AcPIM2 is further modified by two additional mannoses, which are added by an unknown mannosyltransferase(s), resulting in the formation of AcPIM4. AcPIM4 is the branching point between AcPIM6 and LM/LAM biosynthesis. PimE, an α1,2 mannosyltransferase, adds the fifth mannose from polyprenol phosphate mannose (PPM), committing the pathway to AcPIM6 synthesis (8). AcPIM4 feeds into LM/LAM biosynthesis through an unknown mannosyltransferase, which produces an LM intermediate carrying 5 to 12 mannose residues. From there, two PPM-dependent mannosyltransferases are involved in producing mature LM, which carries 21 to 34 mannose residues. MptA, an α1,6 mannosyltransferase, elongates the mannan backbone, while MptC, an α1,2 mannosyltransferase, decorates the α1,6 mannan backbone with α1,2 mono-mannose side chains (18–22). The addition of an arabinan chain consisting of ∼70 arabinose residues to LM finally leads to the production of LAM, and a number of arabinosyl transferases are involved in this process (2, 3, 5).

Previously, we have shown that the conserved protein LmeA is involved in the final maturation steps of LM biosynthesis in M. smegmatis, potentially aiding MptA in mannan elongation (23). Interestingly, in M. tuberculosis, LmeA is predicted to be essential and upregulated upon mouse infection (24–26). In this study, we examined the role of LmeA in M. smegmatis under stress conditions. We demonstrated that LmeA maintains the cellular levels of MptA and that its expression level correlates with the cellular LM content and cell envelope integrity.

## RESULTS

### An M. tuberculosis ortholog is functional in M. smegmatis.

**FIG 1:**
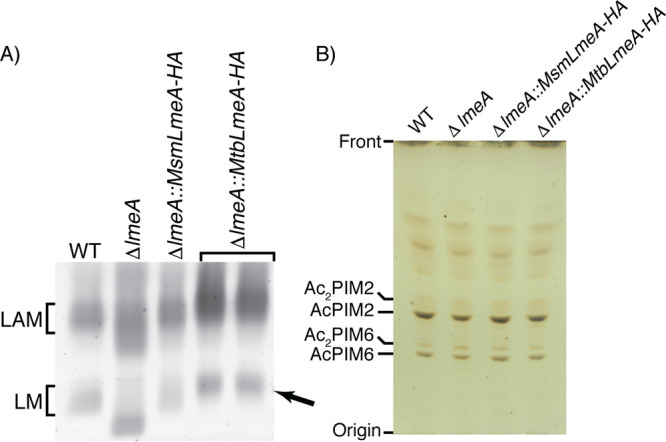
M. tuberculosis LmeA is the functional ortholog of M. smegmatis LmeA. The Δ*lmeA* M. smegmatis strain was complemented with the M. tuberculosis LmeA ortholog (*rv0817c*) expressed at the mycobacteriophage L5 *attB* site. (A) SDS-PAGE analysis of LM and LAM. Two independent clones of the Δ*lmeA*::*Mtb_lmeA* strain were able to restore mature LM formation (arrow). (B) HPTLC analysis of PIMs. PIM profiles were not affected by complementation of *Mtb_lmeA-HA*.

LmeA is a conserved protein in actinobacteria (23). In M. tuberculosis, a protein that is 60% homologous to M. smegmatis LmeA at the amino acid level is encoded in the gene locus *rv0817c*. The expression of this gene is upregulated in M. tuberculosis upon mouse infection (26), implying that LmeA is crucial for the pathogenesis. To validate that Rv0817c is the functional ortholog in M. tuberculosis, we cloned the gene and expressed it in an M. smegmatis *lmeA* deletion (Δ*lmeA*) mutant. As shown previously (23), the Δ*lmeA* mutant accumulated small LM and highly heterogenous LAM, and the M. smegmatis *lmeA* gene complemented the mutant phenotype (Fig. 1A). When the M. tuberculosis homolog (*rv0817c*) was introduced into the deletion mutant instead of M. smegmatis *lmeA*, it was able to restore the LM/LAM phenotype of M. smegmatis Δ*lmeA* (Fig. 1A). LmeA does not have an impact on the biosynthesis of structurally related PIM species, and as expected, M. tuberculosis LmeA showed no effect on their biosynthesis (Fig. 1B). These data suggested that Rv0817c is functionally equivalent to M. smegmatis LmeA. Therefore, M. tuberculosis LmeA, which has been reported to be critical for growth and is upregulated upon mouse infection (24–26), is a functional ortholog, suggesting its involvement in mannan elongation in M. tuberculosis.

### M. smegmatis *lmeA* is upregulated upon stress.

**FIG 2:**
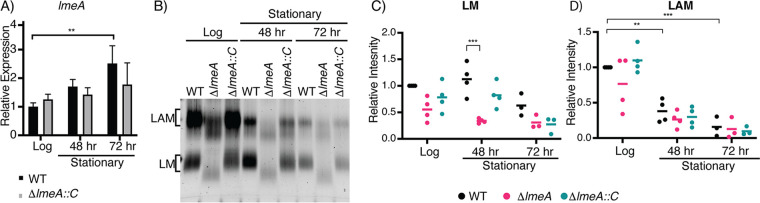
Expression of the *lmeA* transcript and cellular levels of LM/LAM during stationary growth phase. (A) Relative expression of the *lmeA* transcript normalized to that of a housekeeping gene, *gyrB*. A significant increase in *lmeA* expression is noted by 72 h of growth in comparison to that at log phase (18 h). **, *P* < 0.05, by ANOVA. (B) SDS-PAGE analysis of LM/LAM during stationary phase. A representative image of a biological triplicate is shown. LAM decreased significantly more in the stationary phase than LM did. (C and D) Dot plots showing intensities of LM (C) and LAM (D) from biological replicates. Signal intensities were quantified using ImageQuant and normalized to the intensity of the WT at log phase. The averages are shown as horizontal bars. **, *P* < 0.01; *, *P* < 0.05, by ANOVA. Δ*lmeA*::*C*, Δ*lmeA*::P_native_*lmeA-HA* strain (also see the text).

The upregulation of M. tuberculosis *lmeA* (*rv0817c*) during mouse infection implies that expression of this gene may be a response to environmental stress. Given that Rv0817c is orthologous to M. smegmatis LmeA, we speculated that LmeA may respond to stress conditions in M. smegmatis as well. To examine if *lmeA* gene expression is upregulated upon stress, we extracted RNA from cells under stress and examined the transcript levels of *lmeA* in M. smegmatis strains (the wild type [WT], Δ*lmeA* mutant, and Δ*lmeA*::P_native_*lmeA-HA* strain) by quantitative real-time PCR (qRT-PCR). As a model stress condition, we first tested the stationary growth phase and found that *lmeA* was induced 2.5-fold by 72 h in WT cells compared to levels at log phase (Fig. 2A). In addition, the Δ*lmeA* mutant complemented with the *lmeA* gene, which carries its upstream 165 bp as a putative endogenous promoter region (Δ*lmeA*::P_native_*lmeA-HA*) (23), showed a similar trend of upregulation, although the upregulation was not statistically significant.

The cellular levels of LAM rapidly decline in stationary phase, but the levels of LM remain relatively constant regardless of the growth phase (21, 27). Because LmeA plays a role in mannan biosynthesis (23), we speculated that the deletion of *lmeA* may affect LM maintenance during stationary phase. As with previous studies, we noted a 2.5-fold decrease in LAM levels in the WT by the 72-h time point, and similar declines in LAM levels were observed in the Δ*lmeA* and Δ*lmeA*::P_native_*lmeA-HA* strains in the stationary phase (Fig. 2B and D; see also Fig. S1A and S1B in the supplemental material). In contrast, the LM levels of WT cells were not significantly different between log and stationary phases (Fig. 2B and C; Fig. S1A and S1B). Strikingly, LM levels in the Δ*lmeA* mutant were 3-fold lower than those in the WT at the 48-h time point (Fig. 2C). The observed decline in LM levels in the Δ*lmeA* mutant is specific, as there were no specific changes in the levels of other lipids, such as phospholipids and PIMs, that can be attributable to the lack of LmeA (Fig. S1C). Overall, the loss of LmeA not only makes mannan elongation defective but also impacts the maintenance of LM levels during stationary phase.

10.1128/mSphere.01039-20.2 FIG S1 Phospholipid analysis of stationary-phase cells. (A and B) Two additional replicates of SDS-PAGE analysis of LM/LAM purified from growth phase time points. (C) HPTLC analysis of lipids purified from growth phase time points. (Top) Iodine staining; (bottom) orcinol staining. PI, phosphatidylinositol; CL, cardiolipin; PE, phosphatidylethanolamine. A slight increase in AcPIM2 is noted in stationary phase (48 and 72 h). There were no significant differences among the WT, Δ*lmeA*, and Δ*lmeA*::P_native_*lmeA-HA* strains (indicated as Δ*lmeA*::*C*). Download FIG S1, TIF file, 1.5 MB. Copyright © 2020 Rahlwes et al. 2020 Rahlwes et al. This content is distributed under the terms of the Creative Commons Attribution 4.0 International license.

### LmeA is critical for the maintenance of MptA during stress.

**FIG 3:**
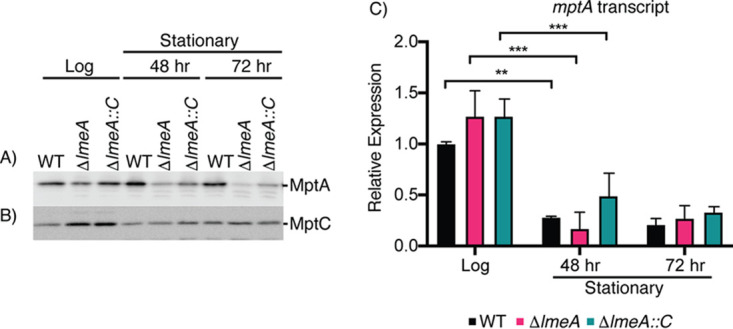
Protein levels of MptA decrease significantly in the Δ*lmeA* mutant during stationary phase. (A and B) Western blot analysis of MptA (A) and MptC (B) over a 72-h time course of growth. MptA shows a significant decrease in the Δ*lmeA* mutant. MptC remains fairly constant between the WT, Δ*lmeA* mutant, and Δ*lmeA*::P_native_*lmeA-HA* strain. For SDS-PAGE, the same concentration of protein was loaded into each lane. (C) Relative expression levels of the *mptA* transcript were normalized to that of *gyrB*. A dramatic decrease in mRNA transcript levels is noted between log phase and 48 h (stationary phase). ***, *P* < 0.001; **, *P* < 0.05, by ANOVA. At each time point, there were no significant differences among the WT, Δ*lmeA* mutant, and Δ*lmeA*::P_native_*lmeA-HA* strain. Δ*lmeA*::*C*, Δ*lmeA*::P_native_*lmeA-HA* strain.

Since LmeA is involved in a step of LM biosynthesis at or near the MptA-mediated mannan elongation (23), we speculated that the lack of LmeA may affect MptA abundance, leading to the decreased amount of LM during stationary phase. To examine this possibility, we examined the MptA protein levels in anti-MptA Western blots at each time point (Fig. 3A). The abundance of MptA was maintained at similar levels during the stationary phase in WT cells. In contrast, in the Δ*lmeA* mutant, the protein level was reduced at both the 48- and 72-h time points, and these reductions in MptA abundance were alleviated in the complemented strain. To confirm that this decrease is not a general reduction in protein abundance, we examined changes in the abundance of another mannosyltransferase, MptC, which mediates the α1,2 mono-mannose addition. The levels of MptC were comparable among the three strains. There were no specific changes in its abundance that can be attributed to the *lmeA* gene deletion (Fig. 3B). We therefore conclude that LmeA is necessary for the maintenance of MptA protein.

Is the reduced abundance of MptA protein in Δ*lmeA* mutant stationary-phase cells due to downregulation of *mptA* transcription in the mutant? To answer this question, we determined the relative levels of the *mptA* transcript by qRT-PCR. While there were no significant differences in the levels of expression of *mptA* between the three strains, a general decrease was observed in *mptA* transcript levels at 48 h in comparison to levels in the log-phase cells (Fig. 3C). After this time point, the transcript levels of *mptA* were stable, with no further decrease. The result that mRNA levels were comparable between the WT and Δl*meA* mutant suggests that the cellular MptA level declines at the protein level during stress conditions in the absence of LmeA.

**FIG 4:**
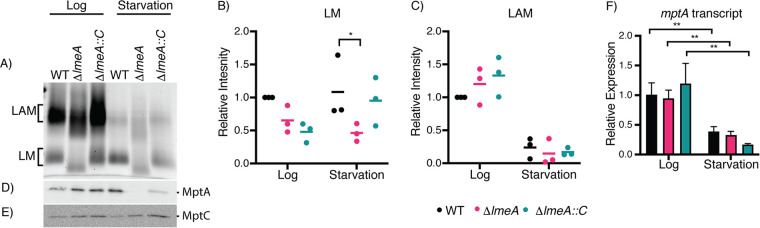
Effect of starvation on cellular levels of LM/LAM and their biosynthetic enzymes. (A to C) SDS-PAGE analysis of LM/LAM. LAM decreases during starvation, while LM remains constant. A representative image of biological triplicate is shown. LM (B) and LAM (C) from a biological triplicate were quantified, and changes relative to WT levels in log phase are shown in dot plots. Averages are shown as horizontal bars. (D) Western blot analysis of MptA in log phase and after starvation. MptA is decreased in the Δ*lmeA* mutant in comparison to levels in the WT during starvation. (E) Western blot of MptC in log phase and after starvation. No significant decrease was noted among the WT, Δ*lmeA* mutant, and Δ*lmeA*::P_native_*lmeA-HA* strain before and after starvation. Equal amounts of protein were loaded for Western blot analysis. (F) Transcript levels of *mptA* relative to levels in the WT in log phase, normalized to *gyrB* levels. The levels of the *mptA* transcript decreased from log phase to starvation, but deletion of the *lmeA* gene had no apparent impact. **, *P* < 0.01, by ANOVA. Δ*lmeA*::*C*, Δ*lmeA*::P_native_*lmeA-HA* strain.

Next, we used starvation as another condition to characterize stress on LM/LAM biosynthesis. We starved M. smegmatis strains (the WT, Δ*lmeA* mutant, and Δ*lmeA*::P_native_*lmeA-HA* strain) in phosphate-buffered saline (PBS) for 24 h. We found an increase in *lmeA* transcripts upon starvation, which was also observed in the Δ*lmeA*::P_native_*lmeA-HA* strain (Fig. S2A). As with the trend of transcriptional upregulation in the complemented strain, we detected a higher level of LmeA-HA protein when the Δ*lmeA*::P_native_*lmeA-HA* strain was starved (Fig. S2B). As observed in stationary-phase cells, there was a substantial depletion of LAM upon starvation, while LM remained relatively constant in the WT (Fig. 4A, B, and C; Fig. S3A and S3B). As with the observed depletion of LM in the stationary-phase Δ*lmeA* mutant, LM abundance in the Δ*lmeA* mutant during starvation was at ∼50% of the level in the WT. The abundances of other membrane phospholipids appeared similar among the three strains (Fig. S3C). Furthermore, MptA protein levels were ∼20 times lower in the Δ*lmeA* mutant than in the WT, a more dramatic fold change than that of stationary phase. In contrast, changes in MptC levels were much milder, being less than 2-fold (Fig. 4D and E). The Δ*lmeA*::P_native_*lmeA* strain was able to partially complement the phenotype, although MptA levels were 3.5-fold less than that in the WT. These changes in MptA are at the protein level because the *mptA* transcript did not show specific changes in the Δ*lmeA* mutant (Fig. 4F). Overall, starvation is another stress condition where *lmeA* gene expression is upregulated, and the presence of LmeA is critical for the maintenance of MptA and LM.

10.1128/mSphere.01039-20.3 FIG S2 LmeA expression during starvation. (A) Expression of the *lmeA* transcript in the WT and Δ*lmeA*::P_native_*lmeA-HA* strains upon starvation. The *lmeA* expression levels were normalized to those of *gyrB* and are graphed relative to the expression level of WT log-phase cells. (B) Anti-HA Western blot of the Δ*lmeA*::P_native_*lmeA-HA* strain during active growth and starvation. A slight increase in LmeA-HA expression was noted. Download FIG S2, TIF file, 0.8 MB. Copyright © 2020 Rahlwes et al. 2020 Rahlwes et al. This content is distributed under the terms of the Creative Commons Attribution 4.0 International license.

10.1128/mSphere.01039-20.4 FIG S3 Phospholipid analysis of starved cells. (A and B) Two additional replicates of SDS-PAGE analysis of LM/LAM purified from log-phase and starved cells. (C) HPTLC analysis of lipids purified from log-phase and starved cells. (Top) iodine staining; (bottom) orcinol staining. There were no differences that can be attributed to the lack of LmeA. Δ*lmeA*::*C*, Δ*lmeA*::P_native_*lmeA-HA* strain. Download FIG S3, TIF file, 1.4 MB. Copyright © 2020 Rahlwes et al. 2020 Rahlwes et al. This content is distributed under the terms of the Creative Commons Attribution 4.0 International license.

**FIG 5:**
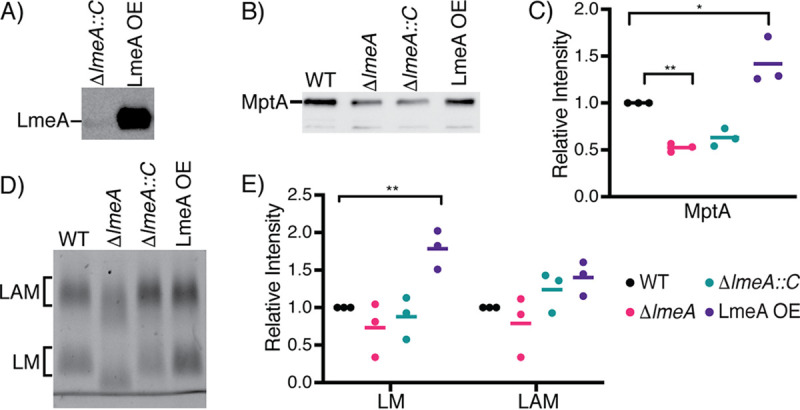
Impact of overexpression of LmeA on LM/LAM biosynthesis. (A) Anti-HA Western blot to detect LmeA-HA expressed in the Δ*lmeA*::P_native_*lmeA-HA* strain (indicated in the figure as Δ*lmeA*::*C*) and LmeA-HA overexpression strain (LmeA OE). LmeA-HA was ∼10-fold more intense in LmeA OE than in the Δ*lmeA*::P_native_*lmeA-HA* strain. (B) Western blot analysis of MptA in the actively growing WT, Δ*lmeA*, Δ*lmeA*::P_native_*lmeA-HA*, and LmeA-HA OE strains. The same amount of protein was loaded into each lane. A representative image of a biological triplicate is shown. Δ*lmeA*::*C*, the Δ*lmeA*::P_native_*lmeA-HA* strain. (C) Dot plot showing relative abundances of MptA in a biological triplicate. Intensity was normalized to that of the WT, and the average is shown as a horizontal bar. *, *P* < 0.05; **, *P* < 0.01, by ANOVA. (D) Cellular levels of LM and LAM in the actively growing WT, Δ*lmeA*, Δ*lmeA*::P_native_*lmeA-HA*, and LmeA OE strains. A representative image of three biological replicates is shown. (E) Dot plot of cellular LM/LAM levels. The average from biological replicates is shown as a horizontal bar. **, *P* < 0.01, by ANOVA.

Given that *lmeA* transcription was upregulated upon stress exposure and that the deletion of *lmeA* resulted in decreased abundances of LM both in stationary phase and under starvation, we considered the possibility that the overexpression of LmeA may impact LM abundance even during active growth. We created an episomal expression vector for LmeA-hemagglutinin (HA), in which gene expression is driven by the strong heat shock protein 60 (HSP60) promoter (28). In this LmeA overexpression strain (LmeA OE), LmeA-HA protein levels were ∼10-fold higher than those in the Δ*lmeA*::P_native_*lmeA-HA* strain, where *lmeA-HA* transcription is driven by the putative endogenous promoter (Fig. 5A). In response to the overexpression of LmeA-HA, MptA levels show an increase of ∼40% in LmeA OE compared to those in the WT (Fig. 5B and C). Concomitantly with the increase in MptA, LM levels were increased by ∼80% (Fig. 5D and E; Fig. S4A and S4B). In contrast, in the Δ*lmeA* mutant, the MptA levels decreased by ∼1.9-fold compared to those in the WT even in the log phase. The impact of LmeA OE is specific to LM, as it does not show a significant impact on LAM levels and does not impact the abundance of PIMs and other phospholipids (Fig. S4C). Taken together, these data suggest that LmeA not only prevents the reduction of MptA under stress conditions but also helps to enhance the production of LM during active growth.

10.1128/mSphere.01039-20.5 FIG S4 Phospholipid analysis of cells overexpressing LmeA. (A and B) Two additional replicates of SDS-PAGE analysis of LM/LAM-purified LmeA OE and other strains. (C) HPTLC analysis of lipids purified from LmeA OE and other strains. (Top) Iodine staining; (bottom) orcinol staining. There were no differences that can be attributed to the lack of LmeA. Δ*lmeA*::*C*, Δ*lmeA*::P_native_*lmeA-HA* strain. Download FIG S4, TIF file, 1.1 MB. Copyright © 2020 Rahlwes et al. 2020 Rahlwes et al. This content is distributed under the terms of the Creative Commons Attribution 4.0 International license.

### The expression level of LmeA correlates with antibiotic resistance.

Previously, by use of *mptA* knockdown and *mptC* deletion mutants, LM/LAM were shown to play important roles in conferring intrinsic resistance to various antibiotics (10, 21). Given the significant impacts of *lmeA* deletion and overexpression on LM/LAM as shown above and previously (23), we examined the impact of these changes in LM/LAM on antibiotic resistance. We determined the MIC_90_s of five antibiotics that target either periplasmic PG biosynthesis (ampicillin, cefotaxime, and vancomycin) or cytoplasmic protein synthesis (clarithromycin and erythromycin) (Table 1). The Δ*lmeA* mutant was more sensitive to all five antibiotics than the WT. In particular, the MIC_90_s of cefotaxime and erythromycin for the Δ*lmeA* mutant were >10- and 8-fold reduced in comparison to those for the WT, respectively. The complementation of the mutant (Δ*lmeA*::P_native_*lmeA*) restored resistance to all antibiotics, but the resistance to vancomycin, cefotaxime, and erythromycin was only partially restored. As we demonstrated previously that antibiotic sensitivity is dependent on the growth medium (9), we further tested sensitivities to these antibiotics in a medium different from Middlebrook 7H9, our standard growth medium. As shown in Table 2, WT M. smegmatis grown in M63 medium became notably more resistant to vancomycin than strains grown in Middlebrook 7H9. The MIC_90_s of clarithromycin and erythromycin also became higher in M63 than in Middlebrook 7H9. Nevertheless, the increased sensitivities to antibiotics found in the Δ*lmeA* mutant were reproducible in M63, and the complemented strain restored antibiotic resistance at least partially (Table 2). Taken together, the Δ*lmeA* mutant is more sensitive to various antibiotics, likely due to its truncated mannan backbone of LM and LAM compromising the cell envelope integrity.

**TABLE 1:** Antibiotic sensitivities of M. smegmatis strains grown in Middlebrook 7H9 medium

Strain	MIC_90_ (μg/ml)^*a*^ of:				
	Vancomycin	Cefotaxime	Ampicillin	Clarithromycin	Erythromycin
WT	1.00 ± 0.14	>100	>100	0.15 ± 0.01	0.96 ± 0.17
Δ*lmeA* mutant	0.39 ± 0.04	9.77 ± 2.08	70.0 ± 14.8	0.05 ± 0.01	0.12 ± 0.01
Δ*lmeA*::P_native_ *lmeA-HA* strain	0.58 ± 0.09	48.2 ± 7.6	>100	0.15 ± 0.01	0.31 ± 0.01
LmeA OE	2.13 ± 0.33	>100	>100	0.25 ± 0.05	0.92 ± 0.21

a Values are averages ± standard deviations.

**TABLE 2:** Antibiotic sensitivities of M. smegmatis strains grown in M63 medium

Strain	MIC_90_ (μg/ml)^*a*^ of:				
	Vancomycin	Cefotaxime	Ampicillin	Clarithromycin	Erythromycin
WT	>100	>100	>100	1.61 ± 0.24	78.9 ± 28.2
Δ*lmeA* mutant	14.7 ± 2.9	>100	>100	0.35 ± 0.03	0.41 ± 0.16
Δ*lmeA*::P_native_*lmeA-HA* strain	99.6 ± 17.9	>100	>100	0.61 ± 0.02	9.51 ± 1.64

a Values are averages ± standard deviations.

LmeA OE, the strain overexpressing LmeA in the WT background, showed enhanced LM abundance compared with that in the WT. We wondered if the increased abundance of LM might impact the antibiotic resistance. As shown in Table 1, LmeA OE showed up to an ∼2-fold increase in resistance to vancomycin and clarithromycin (Table 1), indicating that the amount of LM correlates with antibiotic resistance.

## DISCUSSION

The cellular amount of LAM decreases as M. smegmatis enters stationary phase (21, 27). In contrast, the amount of LM remains relatively stable during the growth phase shift. The molecular mechanisms behind the selective maintenance of cellular LM levels remain unknown. In this study, we examined the role of a periplasmic protein, LmeA, under stress conditions. Our data are consistent with a regulatory mechanism in which the *lmeA* gene is upregulated under stress and in which the increased level of LmeA protein ensures the maintenance of the mannosyltransferase MptA and its product LM.

**FIG 6:**
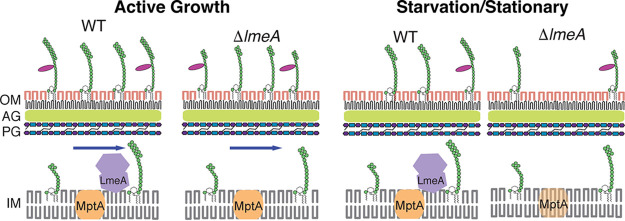
Proposed model of how LmeA functions during stress conditions. Under active growth conditions, LmeA aids MptA in its function, potentially through either maintaining MptA or facilitating the trafficking of LM/LAM or both. Upon stress exposure, such as starvation or stationary phase, LmeA prevents the decline of the cellular MptA level. IM, inner membrane; PG, peptidoglycan; AG, arabinogalactan; OM, outer membrane.

To support our model, we tested nutrient starvation as another stress model in addition to stationary phase and demonstrated that LAM selectively decreases under both stress conditions (Fig. 6). The decrease in the cellular amount of LAM cannot be explained by the depletion of the LM precursor because LM as well as the biosynthetic mannosyltransferases MptA and MptC remain relatively stable under these conditions. We further showed that the maintenance of MptA under stress conditions requires LmeA. MptA is a key mannosyltransferase that mediates the mannan core elongation, and the defective maintenance of MptA in the Δ*lmeA* mutant correlated with a reduction in LM levels. Taken together, LmeA appears to function as a regulator in the stress response, preventing the decline of MptA levels and thereby maintaining LM levels.

Since there is no difference in *mptA* mRNA levels between the Δ*lmeA* mutant and the WT, LmeA maintains the cellular level of the mannosyltransferase MptA, likely by acting at the posttranscriptional level. How does LmeA prevent the decline of MptA during stress conditions? LmeA is a distant homolog of bactericidal/permeability increasing protein (BPI) (23), and like BPI, it has a tubular lipid-binding (TULIP) lipid transfer domain (29). Furthermore, LmeA also carries a Chorein-N domain, which is also involved in lipid transfer (29). Indeed, we have previously shown that M. smegmatis LmeA can bind to phospholipids (23). Although it is beyond the scope of the current study, we speculate that the full elongation of the mannan chain is coupled with the transfer of LM across the periplasmic space. Such a concurrent process of mannan elongation and lipid transfer would explain why LM intermediates with a short mannan chain accumulate in the Δ*lmeA* mutant. We further speculate that, in the absence of LmeA, MptA may be stalled with partially elongated LM intermediates and may be subjected to degradation, particularly during stress conditions.

The role of LmeA under stress exposure may be further inferred by the location of the gene in the genome. The gene encoding LmeA appears to form an operon with a downstream gene encoding a putative thioredoxin, ThiX (MSMEG_5786 in M. smegmatis and Rv0816c in M. tuberculosis H37Rv), and this operon structure is widely conserved in mycobacteria. ThiX has a predicted signal peptide, suggesting that it is a periplasmic thioredoxin. In Gram-negative bacteria, periplasmic thioredoxins are critical for withstanding redox stress (30). Furthermore, upstream of *lmeA* is *glnR* (MSMEG_5784/Rv0818), which is involved in responding to environmental stresses, such as redox stress and nitrogen starvation (31, 32). *glnR* forms an apparent operon with *mshD*, the final enzyme for the synthesis of mycothiol (33), which is a key molecule to regulate the reduced environment in the cytoplasm. These surrounding genes suggest that *lmeA* is situated in a potential regulon for the redox stress response. How LM/LAM might be important for resisting redox stress remains unclear. However, cell envelope rearrangement is critical for mycobacterial cells when shifting from an actively growing phase to a more stressful nongrowing phase (4). LmeA may play an integral role in regulating the cell envelope rearrangement through facilitating the action of the MptA mannosyltransferase.

Extending previous studies (21, 27), our current study shows that LAM rapidly depletes under nutrient starvation, whereas the abundance of LM does not change significantly. These differences in stress response suggest that LM and LAM play functionally different roles during stress exposures. It remains to be examined if LAM is degraded or shed from the cell under these stress conditions. In either case, LM may play a more vital role in the stabilization of the cell envelope under stress conditions. Indeed, LmeA OE showed an increase in LM but not LAM (Fig. 5B), and this increase in LM was correlated with increased antibiotic resistance (Table 1). Rather subtle changes in the structure and abundance of LM and LAM have a significant impact on cell envelope integrity. We propose that LmeA is a stress response protein that plays a critical role in the biosynthesis and trafficking of LM/LAM, thereby impacting their cell envelope integrity and antibiotic resistance.

## MATERIALS AND METHODS

### Growth conditions.

Mycobacterium smegmatis mc^2^155 (WT), the Δ*lmeA* mutant, the Δ*lmeA*::P_native_*lmeA-HA* strain, the Δ*lmeA*::P_hsp60_*lmeA-HA* strain (23), the Δ*lmeA*::*Mtb_lmeA-HA* strain (where *Mtb* indicates M. tuberculosis) (this study), and LmeA OE (this study) were grown in a liquid medium of Middlebrook 7H9 base (BD) supplemented with glycerol (0.2%, vol/vol), glucose (0.2%, wt/vol), NaCl (15 mM), and Tween 80 (0.05%, vol/vol) at 30°C with shaking at 130 rpm. For analysis, 25 optical density at 600 nm (OD_600_) units of cells were centrifuged for 10 min at 3,220 × *g* and pellets were processed for bead beating, lipid extraction, or RNA extraction.

For the starvation condition, the M. smegmatis strains were grown in Middlebrook 7H9 at 30°C with shaking at 130 rpm until a logarithmic growth phase (OD_600_ = 0.6 to 1.0). The cells were then centrifuged for 10 min at 3,220 × *g*, resuspended in PBS, and incubated for additional 24 h under the same conditions.

### Expression vectors.

Two vectors, the Mtb_LmeA expression vector (pMUM105) and LmeA overexpression vector (pMUM063), were constructed as detailed below. For pMUM063, *lmeA* was excised from pMUM054 (23) by digestion with Van91I and XbaI and ligated into pYAB017 (34), which was digested with Van91I, XbaI, and SphI. pYAB017 has an *oriM* gene and does not integrate into the mycobacterial chromosome. After ligation, the plasmid was electroporated into WT M. smegmatis to create the LmeA overexpression strain. For pMUM105, the gene encoding Rv0817c was PCR amplified from M. tuberculosis genomic DNA using primers A367 and A368 (see Table S1 in the supplemental material). The fragment was then ligated into pDO23A (35) through Gateway BP cloning, creating plasmid pMUM089. The attB-Rv0817c region of this plasmid was then amplified with primers A444 and A445. The PCR fragment was then inserted into pMUM082, which was linearized by ClaI and NdeI, through Gibson assembly. The final plasmid construct, pMUM105, was electroporated into M. smegmatis Δ*lmeA.*

10.1128/mSphere.01039-20.1 TABLE S1 Primers used in this study. See the text for details. Download Table S1, DOCX file, 0.04 MB. Copyright © 2020 Rahlwes et al. 2020 Rahlwes et al. This content is distributed under the terms of the Creative Commons Attribution 4.0 International license.

### Lipid/LM/LAM extraction.

Lipids were extracted as described previously (23, 36). Briefly, lipids were extracted from cell pellets with chloroform-methanol-water and purified by *n*-butanol–water phase partition. Phospholipids and PIMs were analyzed by high-performance thin-layer chromatography (HPTLC) (Silica Gel 60; EMD Merck) using chloroform-methanol-13 M ammonia-1 M ammonium acetate-water (180:140:9:9:23) as the mobile phase. Phospholipids were visualized with iodine, and PIMs were visualized with orcinol as described previously (23, 36). LM and LAM were extracted from the delipidated pellets, separated by SDS-PAGE (15% gel), and visualized using a ProQ Emerald 488 glycan staining kit (Life Technologies) as described previously (23, 36). Intensities of LM and LAM were determined using ImageQuant. Statistical significance was determined by two-way analysis of variance (ANOVA) using GraphPad Prism version 8.

### Cell lysis and protein analysis.

Cell pellets were lysed by bead beating as previously described (23). The protein concentration of each lysate was determined with a bicinchoninic acid (BCA) assay kit (ThermoFisher Scientific) and adjusted to 1.2 mg/ml. The lysate (12 μl) was mixed with reducing sample loading buffer, denatured on ice for 30 min, loaded onto a 12% SDS-PAGE gel, and electrophoresed at 150 V for 70 min. Western blotting was performed as previously described (21) using rabbit anti-MptA, rabbit anti-MptC, or mouse anti-HA (Millipore-Sigma) at a 1:2,000 dilution as a primary antibody. After incubation with horseradish peroxidase-conjugated anti-rabbit or anti-mouse IgG as a secondary antibody at a 1:2,000 dilution (GE Healthcare.), protein bands were visualized by chemiluminescence and the image was captured with an ImageQuant LAS 4000 mini (GE Healthcare).

### Purification of RNA.

RNA was extracted and purified from the cell lysate as previously described (37). Briefly, cell culture was centrifuged at 3,220 × *g* for 10 min. The wet pellets (∼400 mg) were then frozen at −80°C overnight and then resuspend in 1 ml of TRIzol (ThermoFisher Scientific). Cells were lysed with 0.5 ml of zirconia beads using a BeadBug microtube homogenizer (Benchmark Scientific) at 4°C with beating at 4,000 rpm for 1 min, followed by resting on ice for 2 min. The bead homogenization was performed three times in total and followed by a 5-min incubation at room temperature. The samples were then centrifuged for 30 s at 10,000 × *g* at 4°C. The purified RNA was then concentrated using an RNA Clean and Concentrator kit by following the manufacturer’s protocol (Zymo Research). Two micrograms of RNA was used to synthesize cDNA using Maxima H minus reverse transcriptase (ThermoFisher Scientific) and 1 μM random hexamers (Promega) in accordance with the manufacturer’s instructions.

### Quantitative PCR.

For each 25-μl reaction mixture, 12.5 μl of the Roche FastStart Universal SYBR green master mix (Millipore Sigma) was mixed with cDNA and primers to achieve the final concentrations of 100 ng/μl and 400 nM, respectively (Table S1). The quantitative PCR was performed on a Stratagene Mx3005P apparatus (Agilent Technologies) with the following program: 95°C for 10 min, followed by 40 cycles of incubations at 95°C for 30 s, 60°C for 30 s, and 72°C for 30 s. Data were analyzed to determine the 2^–ΔΔ^*^CT^* (where *CT* is the threshold cycle) using the MxPro software and evaluated for statistical significance through GraphPad Prism version 8.

### Antibiotic sensitivity.

Frozen aliquots of the WT, Δ*lmeA*, Δ*lmeA*::P_native_*lmeA-HA*, Δ*lmeA*::P_hsp60_*lmeA-HA*, and LmeA OE strains were prepared from log-phase cultures (OD_600_ = 0.5 to 1.0) grown in Middlebrook 7H9, and the numbers of CFU were determined. In 96-well microtiter plates, antibiotics were serially diluted in 100 μl of either 7H9 or M63 and mixed with 100 μl of cells from the frozen stocks to achieve the final density of 5.0 × 10^3^ CFU/ml. The plates were incubated in a humidity chamber at 37°C for Middlebrook 7H9 or 30°C for M63. After a 24-h incubation, 20 μl of 0.015% (wt/vol) resazurin solution was added to initiate colorization. After an additional 8 h for Middlebrook 7H9 or 13.5 h for M63 incubation, the plates were read on a spectrophotometer at 570 and 600 nm. The percent difference in cell viability between antibiotic-treated and control cells was calculated as described before (9). The MIC_90_ values were calculated using OriginPro 9.1 data analysis software.
