# Supplementary figures and images for: Role of LmeA, a Mycobacterial Periplasmic Protein, in Maintaining the Mannosyltransferase MptA and Its Product Lipomannan under Stress
Source: mSphere. 2020 Nov 4;5(6):e01039-20. doi: 10.1128/mSphere.01039-20 (PMC7643837; doi:10.1128/mSphere.01039-20)

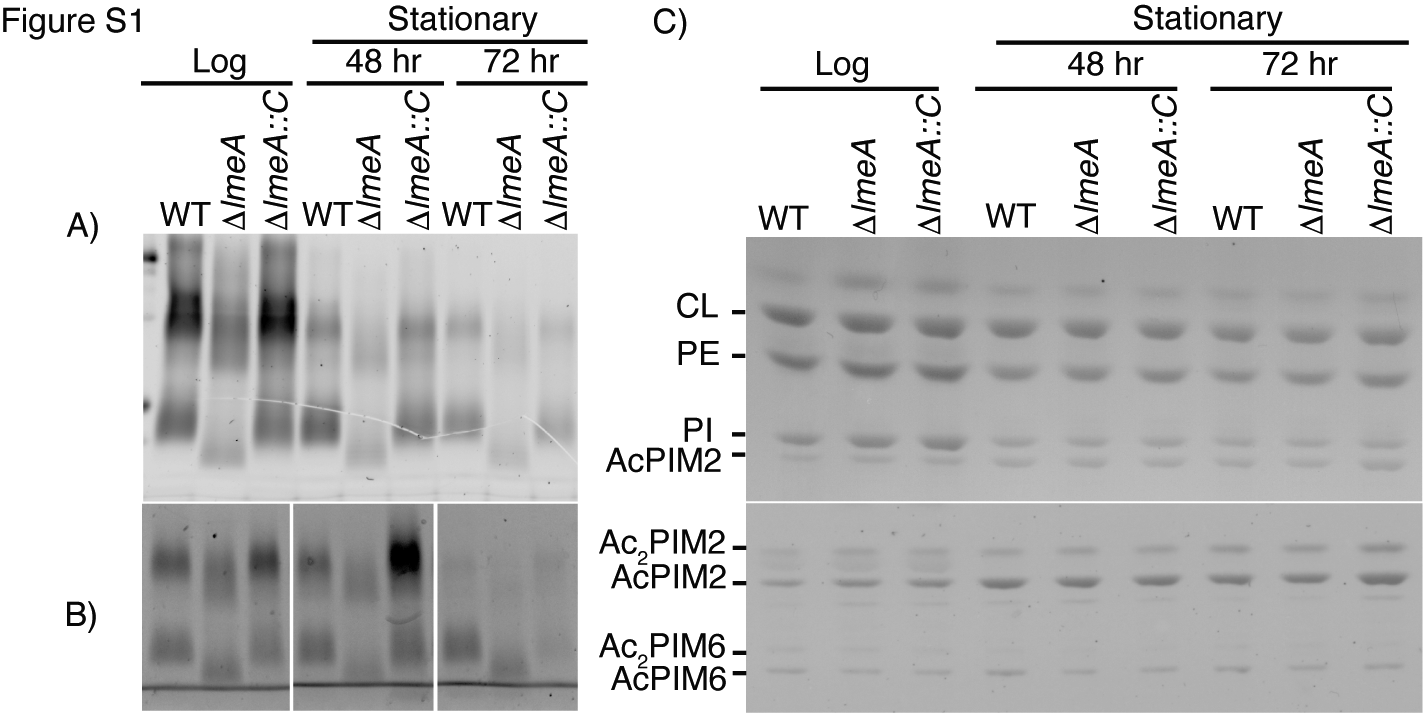

Supplement: FIG S1 [file mSphere.01039-20-sf001.tif]

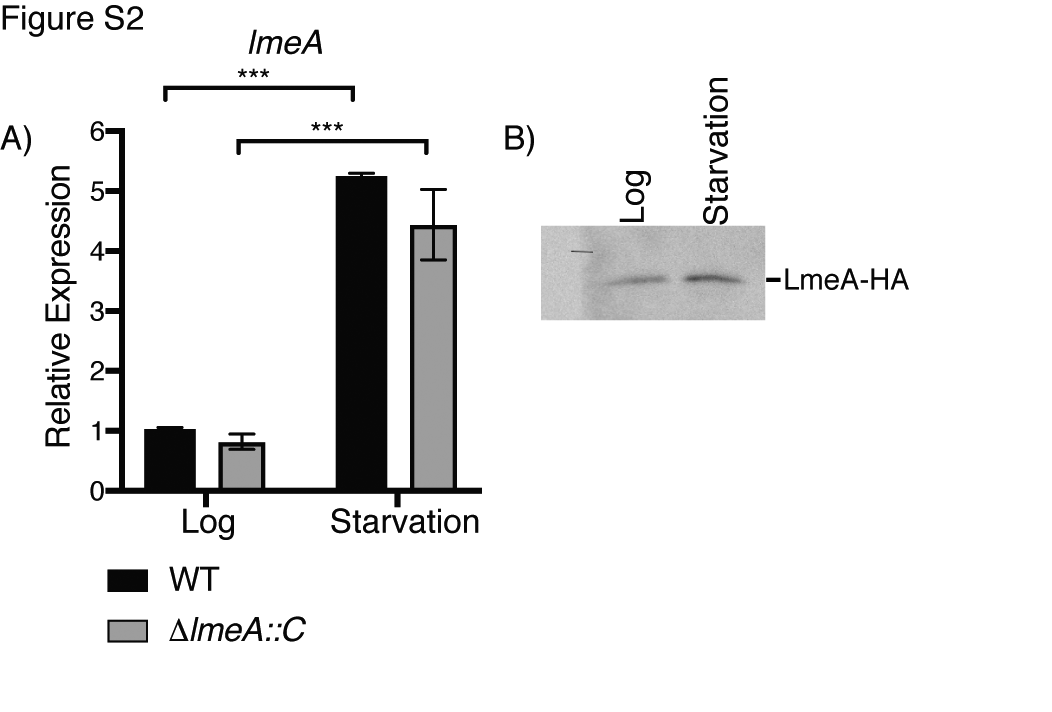

Supplement: FIG S2 [file mSphere.01039-20-sf002.tif]

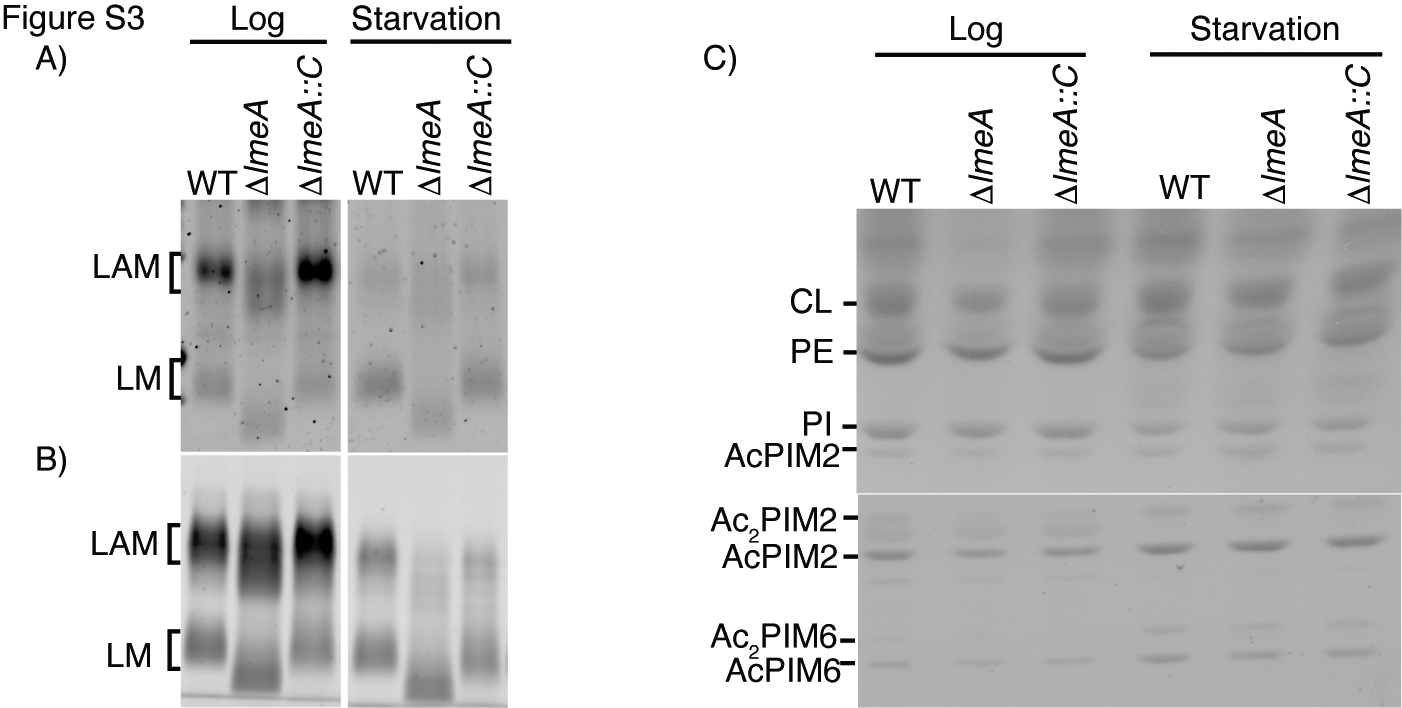

Supplement: FIG S3 [file mSphere.01039-20-sf003.tif]

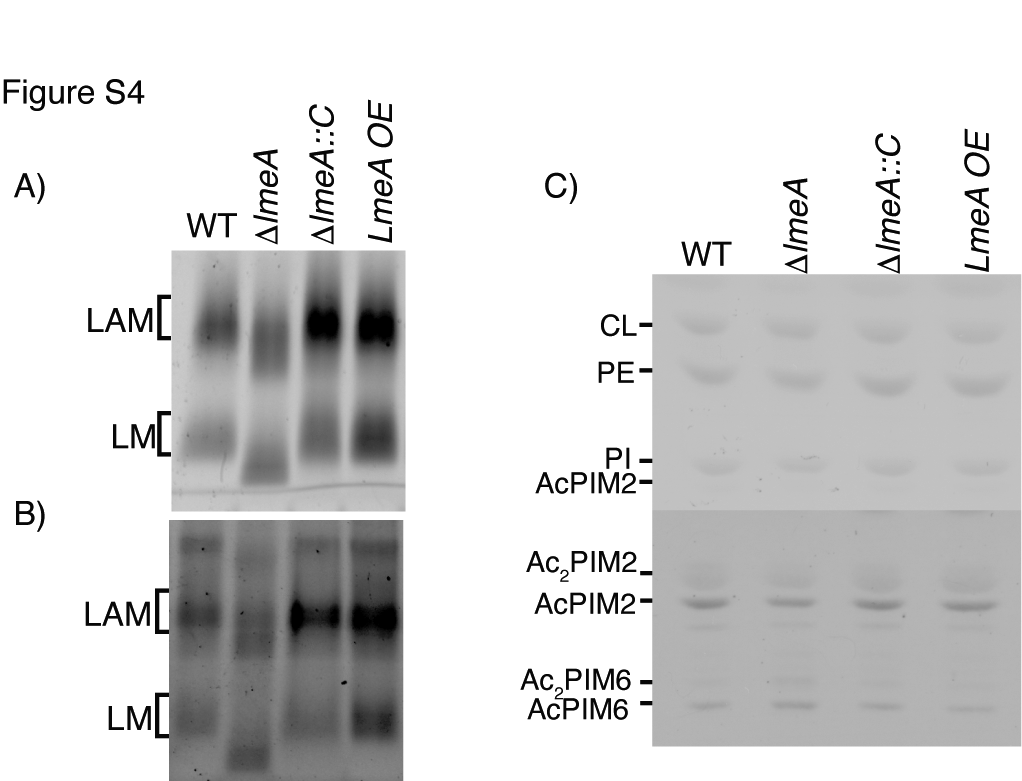

Supplement: FIG S4 [file mSphere.01039-20-sf004.tif]
