# Supplementary material for: Role of LmeA, a Mycobacterial Periplasmic Protein, in Maintaining the Mannosyltransferase MptA and Its Product Lipomannan under Stress
Source: mSphere. 2020 Nov 4;5(6):e01039-20. doi: 10.1128/mSphere.01039-20 (PMC7643837; doi:10.1128/mSphere.01039-20)
Supplement: TABLE S1 [file mSphere.01039-20-st001.docx]

**Supplemental Tables**

**Table S1. Primers used in this study.**

| Primer | Sequence |
| --- | --- |
| A367_attB2_Rv0817c_RBS_CDS_F | GGGGACAGCTTTCTTGTACAAAGTGGACAGAAAGGAGGAAGGAATGCCGATGCGCAAGGTG |
| A368_attB3_Rv0817c_CDS_Stop_R | GGGGACAACTTTGTATAATAAAGTTGCTCATCATGACTGTTTGAACTCGTCGAGCG |
| A444_Gene-Gibson-F | AAGCTTTCTgGTACcAtGgGGACAGAAAGGAGGAAGGAAT |
| A445_Gene-Gibson-R | AGTCGTCGCCACCAATCCCCATATGctcgagtcgcgaattaatGACTGATAGTGACCTGTTCGTTG |
| A711_GyrB F | TATTCGGAGTCGGTGCACAC |
| A712_GyrB R | CTTGTCCTTGGCATACCGGT |
| A715_LmeA F | ATTCAGCAACACCATCCCCG |
| A716_LmeA R | GCCAGGTCGATGGTTACTCC |
| A671_MptA F | TTCAGCCGTGACACGTACTC |
| A657_MptA R | CGGACTCACGTTCTCCAACA |
